# Evaluation of systemic capillary leak syndrome patients with cardiac magnetic resonance imaging

**DOI:** 10.1186/1532-429X-14-S1-O26

**Published:** 2012-02-01

**Authors:** Steve W Leung, Martin Ugander, Peter Kellman, Andrew E Arai, Kirk Druey

**Affiliations:** 1 NHLBI, National Institutes of Health, Bethesda, MD, USA; 2 NIAID, National Institutes of Health, Bethesda, MD, USA

## Description of clinical presentation

Systemic capillary leak syndrome (SCLS) is a rare disorder characterized by transient episodes of hypotension, hemoconcentration and hypoalbuminemia due to leakage of fluids and macromolecules into extracellular space. The generalized edema has been recognized in soft tissue, but has not been described in the myocardium.

## Diagnostic techniques & their most important findings

Patients with systemic capillary leak syndrome and age-matched controls without cardiac disease underwent cardiac magnetic resonance imaging for evaluation of volumes, function, mass, and late gadolinium enhancement. Extracellular volume (ECV) fraction measurements in myocardium were calculated by obtaining T1 values with modified Look-Locker inversion recovery pre-contrast and post contrast injection. Hematocrit was obtained to calculate ECV fraction. Patients with SCLS (n=12) and age-matched controls (n=24) underwent CMR evaluation. Mean age (control: 54±8 years vs. SCLS: 53±9 years, p=0.53), left ventricular ejection fraction (control: 67±6% vs. SCLS: 63±6%, p=0.06), end-diastolic volume indexed to body surface area (control: 75±16ml/m2 vs. 75±18ml/m2, p=0.92) and mass indexed to body surface area (control: 51±10kg/m2 vs. SCLS: 53±11kg/m2, p=0.54) were similar in both groups. There was no significant valvular disorder in either group. Age-matched controls did not have late gadolinium enhancement, whereas one of the patients with SCLS had patchy intermediate atypical late gadolinium enhancement in the inferolateral wall. Five SCLS patients had myocardial ECV fraction greater than 2 standard deviations of the age-matched controls values (Figure). In patients with SCLS, time of last hypotensive episode to imaging ranged from 11 days to 42 months (mean of 15 months), but there was no clear relationship between time of last exacerbation and ECV fraction (r=0.27).

**Figure 1:**
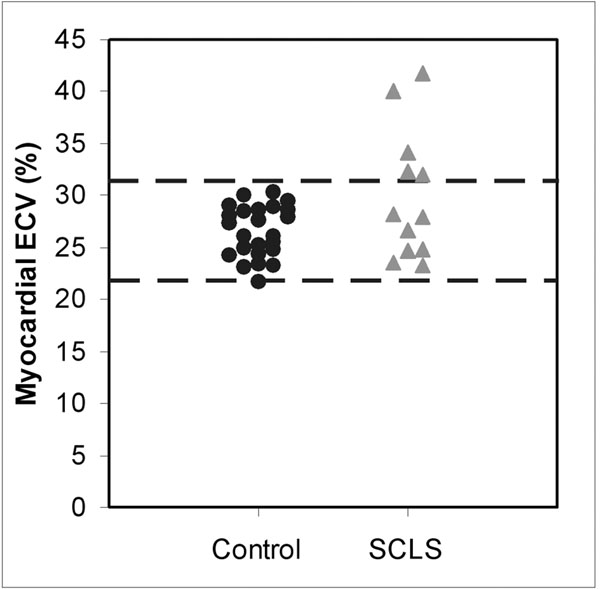


## Learning points from this case

SCLS patients can have significantly elevated myocardial ECV which can be detected by CMR.

Myocardial extracellular volume fraction in age-controlled patients and systemic capillary leak syndrome (SCLS) patients. Five SCLS patients had significantly higher myocardial extracellular fraction compared to age-controlled subjects.

